# Generation of reconfigurable optical traps for microparticles spatial manipulation through dynamic split lens inspired light structures

**DOI:** 10.1038/s41598-018-29540-1

**Published:** 2018-07-26

**Authors:** Angel Lizana, Haolin Zhang, Alex Turpin, Albert Van Eeckhout, Fabian A. Torres-Ruiz, Asticio Vargas, Claudio Ramirez, Francesc Pi, Juan Campos

**Affiliations:** 1 Universitat Autónoma de Barcelona, Physics Department, Optics Group, Bellaterra, 08193 Spain; 2 Center of Advanced European Studies and Research, Bonn, 53175 Germany; 3 Universidad de La Frontera, Departamento de Ciencias Físicas, Temuco, 4811230 Chile; 4 Centro de Ciencias Aplicadas y Desarrollo Tecnológico, Universidad Nacional Autónoma de México, Mexico City, 04510 Mexico

## Abstract

We present an experimental method, based on the use of dynamic split-lens configurations, useful for the trapping and spatial control of microparticles through the photophoretic force. In particular, the concept of split-lens configurations is exploited to experimentally create customized and reconfigurable three-dimensional light structures, in which carbon coated glass microspheres, with sizes in a range of 63–75 *μm*, can be captured. The generation of light spatial structures is performed by properly addressing phase distributions corresponding to different split-lens configurations onto a spatial light modulator (SLM). The use of an SLM allows a dynamic variation of the light structures geometry just by modifying few control parameters of easy physical interpretation. We provide some examples in video format of particle trapping processes. What is more, we also perform further spatial manipulation, by controlling the spatial position of the particles in the axial direction, demonstrating the generation of reconfigurable three-dimensional photophoretic traps for microscopic manipulation of absorbing particles.

## Introduction

Particle trapping methods are unique tools to manipulate and study the microscopic world^1–6^. In the optical context, optical trapping has been exhaustively exploited during the last decades in various physical, biological, and medical applications. The exquisite control over matter provided by optical trapping techniques has served scientists to investigate isolated atoms^7^, molecules^8^, nanoparticles^9–11^, and even cells^12^. The physical mechanisms underlying trapping by optical means are dictated by the optical properties of the particles and the surrounding medium, as well as the physical nature of the light-mediated trapping forces. As an example, standard optical tweezers use the dipole induced by the polarizability of a high-index particle embedded in a low-index medium with the light field to create an attractive trapping potential towards the brightest intensity region. Additional control of particles can be exerted by tailoring the light beam intensity pattern via phase and amplitude modulation or by varying the spatial coherence of light^13–16^.

**Figure 1:**
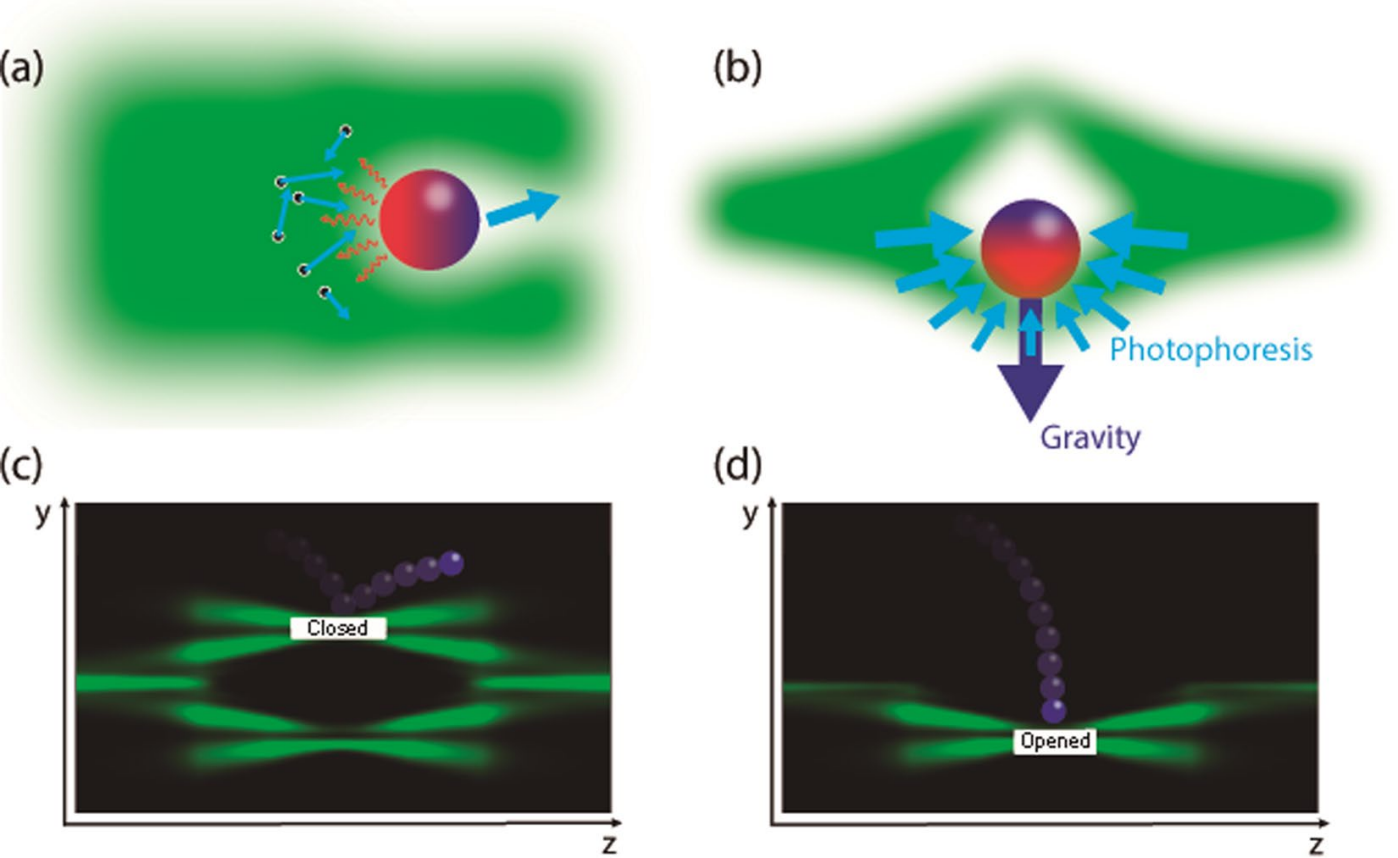
Photophoresis. (**a**) An absorbing particle suspended in a gas medium will receive a net force (blue arrow) due to momentum exchange with the gas molecules as a result of an asymmetric temperature increase. (**b**) If the particle is properly shone, for instance with an optical bottle beam, it can be stably trapped in 3D. (**c**) One of the problems of using bottle beams is that particles are not allowed to penetrate the bottle because of the upper light barrier. (**d**) Optical bottle opened from above permits the loading of particles into it.

For micron-sized-absorbing particles suspended in a gas, the dominant optical force is the photophoretic force^17^, see Fig. 1. Under these conditions, when the particle is illuminated, the energy of the light field rises up the particle temperature. This temperature increase produces heat radiation to the environment (see red arrows in Fig. 1(a)), which also increases the kinetic energy of the gas molecules that, in return, exchanges linear momentum with the particle. This linear momentum exchange results in a net force over the particle (blue arrow in Fig. 1(a)) that pushes it to the opposite direction of the hottest point on the particle surface (see sketch in Fig. 1(a)), this opto-thermal light force being known as photophoresis. In most common situations, the hottest region on the particle surface is the one in direct contact with the light source, as shown in Fig. 1(a), where the colormap of the particle depicts its temperature (from the hottest region -red color- to the coolest region-blue color). In this case, the gas molecules exchanging linear momentum with the particle are non-symmetrically distributed in that region and the direct consequence is that the particle is ejected away from the light source. A very illustrative example is shown in ref.^18^, where the authors demonstrate how the high repellence experienced by absorbing particles from brilliant light regions can be used to design an optical trampoline for microparticles. Similarly, in ref.^19^ the authors use a vortex beam aligned with the gravity field to confine microparticles within the null-intensity region of the vortex and funnel them in the direction of gravity. For efficient photophoretic optical trapping, the gravity and photophoretic forces should be counterbalanced in the vertical direction in order to have a null net force. The simplest method to trap particles via photophortic forces is achieved by using optical bottle beams^20^, which are characterized by possessing an on-axis-null-intensity region surrounded by higher-intensity regions. In Fig. 1(b), we depict photophoretic trapping of an absorbing particle with an optical bottle beam. Along the plane orthogonal to the gravity (horizontal), photophoretic forces are counter-balanced due to the symmetry of the light beam. In the vertical direction, the bottom part of the optical bottle needs to be intense enough to balance the gravity force. As the photophoretic force depends on the shape of the particle, its thermal conductivity, the precise light field distribution, the gas composition, the gas pressure, etc., in experimental situations, gravity is counter balanced by adjusting the power of the trapping light beam until trapping is achieved. The great flexibility offered by photophoretic trapping by means of optical bottle beams has been confirmed in experiments since nearly a decade ago^19,21–25^. Furthermore, photophoretic optical tractor beams have been recently demonstrated by tailoring the state of polarization of the optical bottle^24,26^ and it has also been possible to create a free-space volumetric display by individually addressing the position of cellulose particles in three dimensions^27^. The problem with an ideal bottle beam is that the more efficiently it traps particles the more difficult is to load the bottle with particles. This is because once an optical bottle is formed, it actually prevents particles from penetrate it, as illustrated in Fig. 1(c) and demonstrated in ref.^28^. To cope with this issue, one straightforward solution consists of turning on the bottle beam when the particles already float in the region where the trap will be formed^22^. Another and much more convenient choice would be to design a reconfigurable optical bottle that could be opened/closed allowing one for load/unload particles into/from it, such a mechanism only being demonstrated by means of conical refraction in biaxial crystals^28^, as sketched in Fig. 1(d). Under this approach, the top part of the optical bottle does not repel the particles, which facilitates them to be loaded into the bottom part of the bottle. Once particles are loaded, the bottle can be closed again for better confinement.

In this work we present an alternative method to trap the particles and spatially manipulate micron-sized-absorbing particles through photophoresis. The capture and customized control of particles are achieved by dynamically generating diffractive split-based schemes with a spatial light modulator. The advantage of this technique is the flexibility of the method, as by changing few control parameters of very easy physical interpretation, we are able to create and dynamically modify reconfigurable three-dimensional light structures, this allowing us the fine control of the trapped particles. The potential of the method is experimentally provided by creating light-structures, opening/closing them *ad libitum*, trapping particles, and using the light-capsules to realize 3D reconfigurable photophoretic traps.

The outline of this manuscript is as follows. In the Theoretical background Section, conceptual considerations for the generation of 3D structures through split-lens configurations, and their utilization for the trapping and control of microspheres, according to photophoretic forces, are described. Next, in the Results and Discussion Section, the potential of the method is experimentally demonstrated through a series of experiments related to the experimental generation of customized optical traps, and their use for the trapping and spatial control of microspheres. Afterwards, the main achievements of the work are summarized in the Conclusions Section. Finally, in the Methods Section, we describe the optical arrangement used to experimentally implemented our method, and we provide some commercial characteristics of the optical elements into the system.

## Theoretical Background

The goal of this section is twofold. On the one hand, we provide the basic background related to split-lens configurations, as those schemes are used in this work for the generation of light-based containers where the microspheres are trapped. On the other hand, the generation and control of a particular light structure (light cone) is discussed as a proof-of-concept of the method.

### Split-lens configurations: basic background

**Figure 2:**
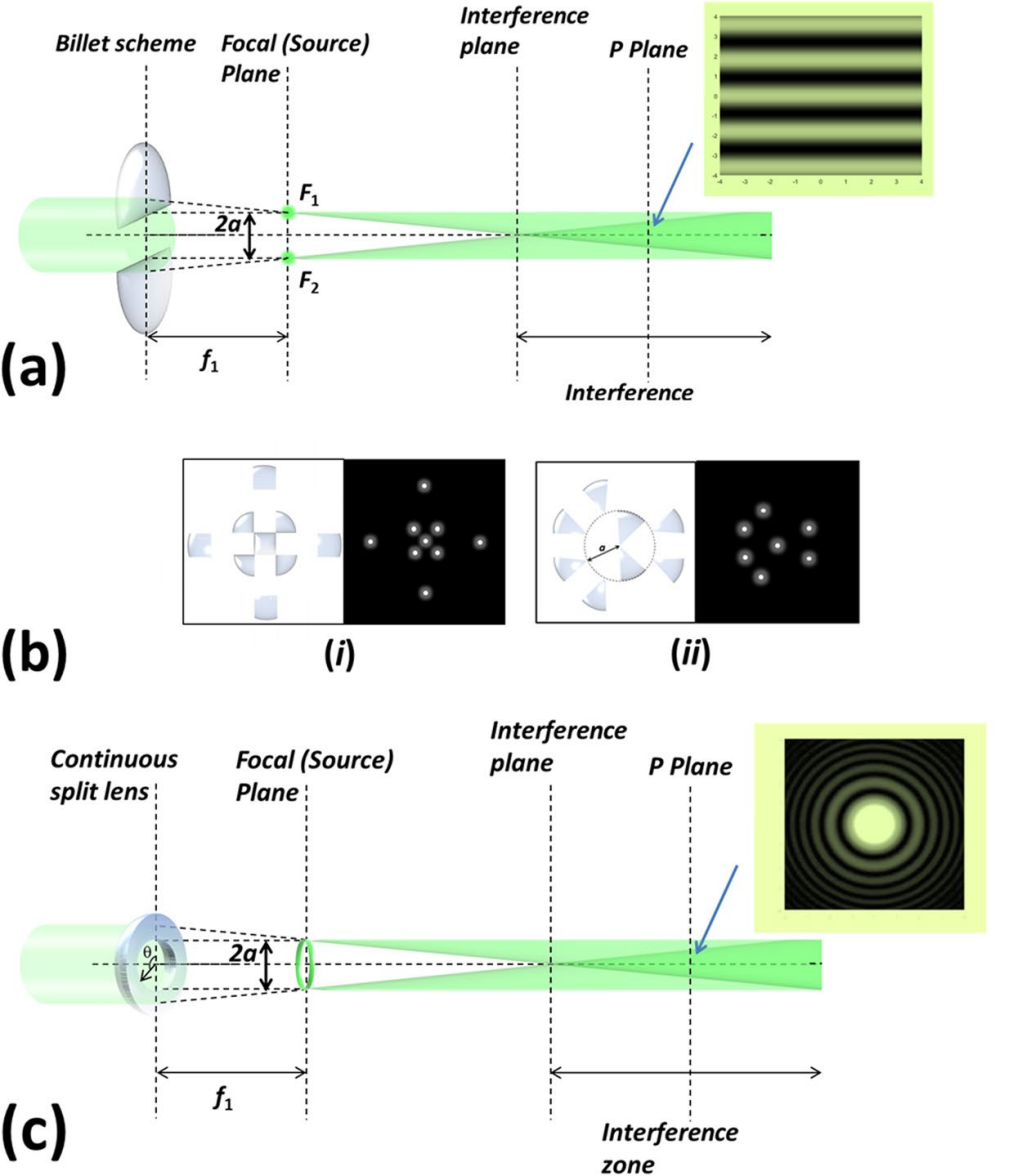
Split-lens configurations proof of concept. (**a**) Two-sectors split lens (classical Billet scheme); (**b**) Two split lens schemes based on two different symmetries: (*i*) Cartesian coordinates; and (*ii*) Polar coordinates; and (**c**) Continuously split lens.

A split-lens configuration is a lens split into *N* different sectors. When illuminating a split-lens scheme, each lens sector focuses the received light in the focal plane, and thus, by controlling the spatial position of different split-lens sectors, a customized light dots distribution can be generated at the focal plane. The dots-distribution control depends on the number *N* of split lens used, as well as on the symmetry used to split the sectors. What is more, these light-dots distribution at the focal plane can be understood as a new (and controlled) light-sources system. As provided in^29^, the propagation of light beams originated from those different light sources leads to two-dimensional and three-dimensional light structures that can be of interest. The first split-lens configuration proposed in literature was the well-known Billet lens (BL) scheme^30,31^, in which a lens is split into two parts (see Fig. 2(a)). Each lens part focuses the input light to a spot at the focal plane, these two spots being separated to a distance equal to the distance between split-lens centers (distance *2a* in Fig. 2(a)). If the system is illuminated with a coherent light, the two focal points becomes into two coherent light sources and thus, an interference pattern is observed to a certain propagated distance. In particular, the Billet-scheme is equivalent to the Young’s experiment, leading to the well-known fringes pattern, as that shown in Fig. 2(a), which corresponds to a given propagated plane *P*. With this BL configuration, the period of the interference fringes can be controlled by modifying the distance *2a* between light sources (F1 and F2 in Fig. 2(a)).

Some authors have shown the interest of generalizing the Billet-lens scheme to *N*-split lens sectors^29,32,33^. The coordinate system chosen to split the lens, strongly influences the light-dots distribution that can be created at the focal plane by using split schemes. As an example, two different particular cases of split-lens schemes based on different coordinates systems are given in Fig. 2(b): (*i*) a lens split in 9 sectors according to the Cartesian coordinate system; and (*ii*) a lens split in 8 sectors according to the Polar system. In both cases, the corresponding light-dots distribution created at the focal plane is also provided. The first mathematical background related to *N*-split lens schemes was provided by C.J. Cheng *et al*.^34,35^, where they also highlighted the potential of using *N* split-lens configurations. Those discrete schemes where recently generalized to continuous schemes^29^. An example of a continuous split-lens scheme is shown in Fig. 2(c). Note that the configuration shown in Fig. 2(c) is achieved by setting a distance to the center *a* common for all the *N* split lens. The flexibility of split-lens schemes allows us to have access to different configurations just by tunning few control parameters. For instance, different 2-D structures, as spiral distributions, can be easily created by selecting a distance to the centers being function of the azimuthal angle *θ*^29^.

The experimental implementation of split-lens configurations carry the difficulty of physically fabricating the different lens sectors with sufficient quality and precision. This is the reason why the first experimental demonstration of split-lens schemes^29^ was based on the use of a Liquid Crystal on Silicon Display^36^ working as a Spatial Light Modulator (SLM), where different experimental split-lens schemes were generated based on diffractive lens addressed to the SLM. The advantages of using diffractive lenses on an SLM over classical split lenses are threefold: First, the generation of the split-lens schemes is easier as we do not need to perform the physical fabrication but only to digitally address the proper phase-only distribution to the SLM. Second, the stray light passing through the holes left between the different split sectors is avoided with the digital implementation, as the phase distribution covers the full modulator screen. Finally, it adds flexibility to the system as different split-lens schemes are dynamically modified just by tunning the proper physical parameters (focal length, lens-sectors distance to the center, etc.).

In this work we use a particular split-lens configuration, the continuous-split lens configuration^29^ to generate the required 3-D light structures intended to capture micro-particles. The mathematical expression for the phase distribution to be addressed at the SLM in order to create a continuous split-lens configuration is provided in Eq. (1). More details related to other configurations can be found in^29^

1 $${U}_{continuouslens}(r,{\theta })=exp[\frac{i\pi }{f\lambda }{(r-a({\theta }))}^{2}],$$

where the argument in Eq. (1) gives the phase to be sent at SLM. The parameters (*r*, *θ*) are the polar coordinates at the lens plane (SLM plane), *λ* gives the wavelength of the light illuminating the system, *f* stands for the continuous split lens focal length and *a*(*θ*) is the split lens to center distance function. Note that if the same displacement *a* is set for all the angles, Eq. (1) no longer depends on *θ*.

Finally, the field propagation corresponding to the complex amplitude given in Eq. (1) can be estimated by using some diffraction integral formula^29^. If doing that, the intensity distribution at the focal plane corresponds to a ring-like intensity distribution with a radius equal to *a*.

### Split-lens schemes for the generation of three-dimensional light structures

**Figure 3:**
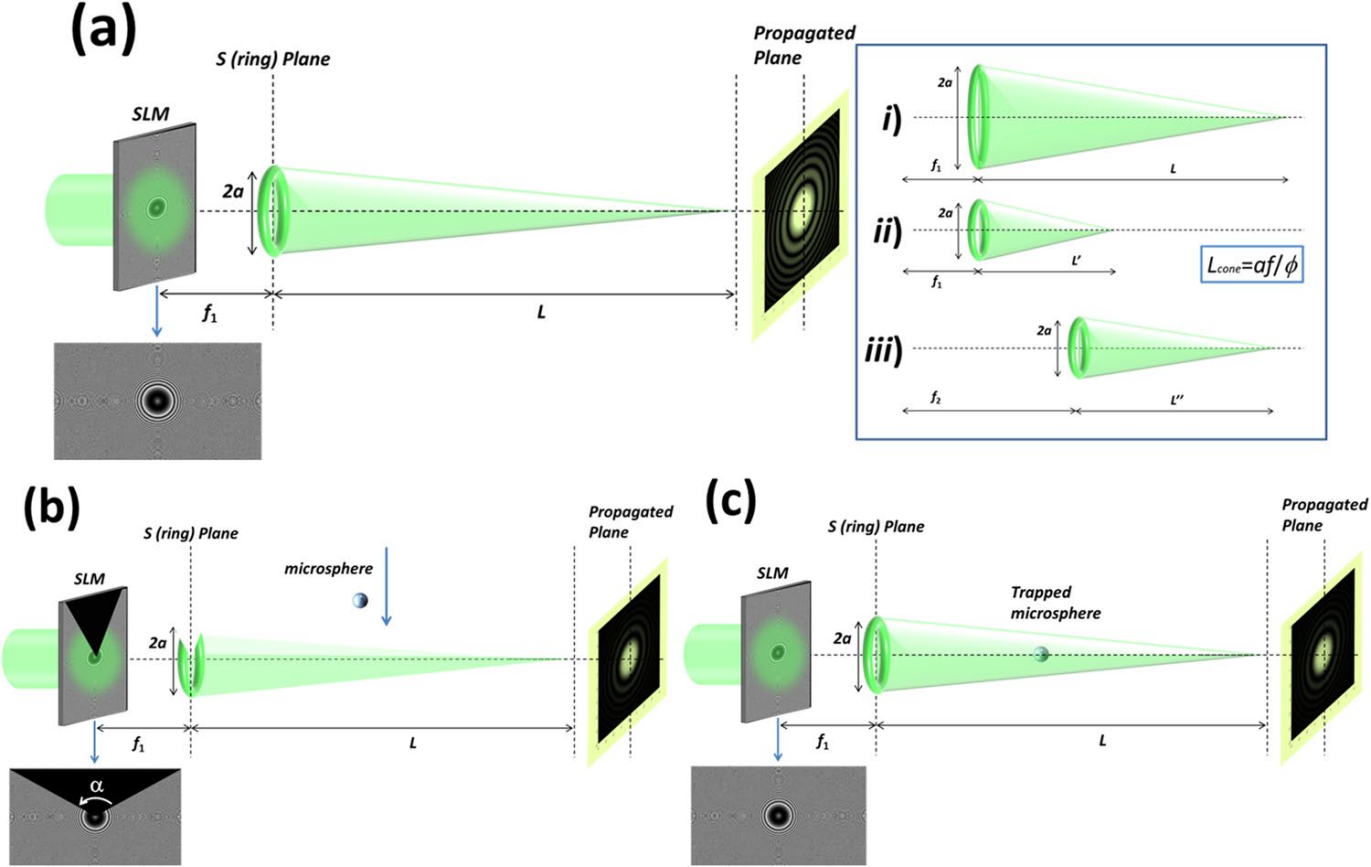
Proof of concept of the method. Light cone example: (**a**) Generation of the light cone; (**b**) Opened light cone and microsphere; and (**c**) Trapping of the particle by closing the light cone. Inset in 3(a); Dependence of the cone geometry with some control parameters: (*i*) Light cone generated for a given split lens shift to the center *a* and focal length *f*; (*ii*) Geometry modification caused by reducing the value of *a*; and (*iii*) Geometry modification caused by increasing the value of *f*.

**Figure 4:**
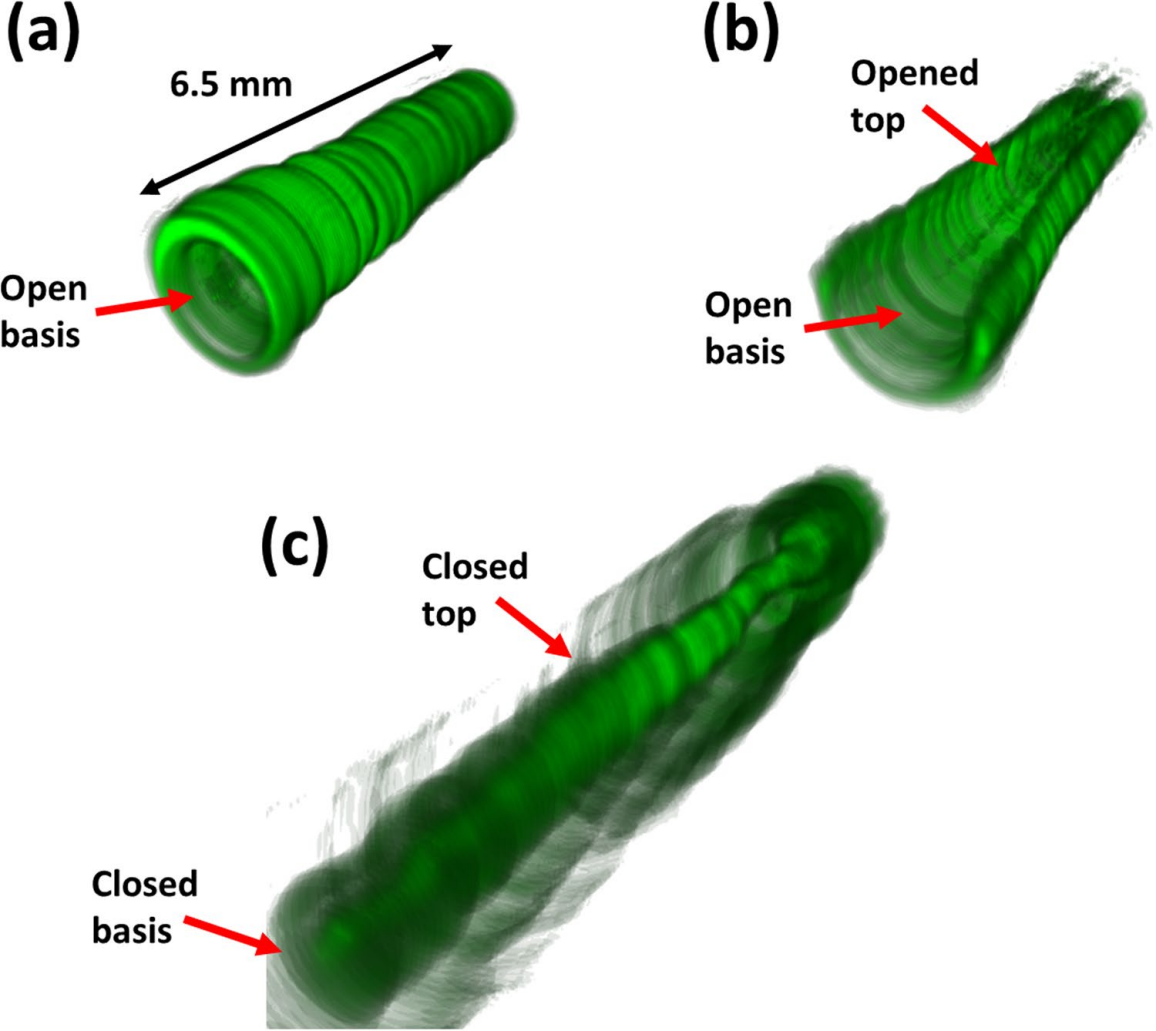
(**a**) Experimentally generated light-cone (closed from the top and opened from the basis); (**b**) Same cone than that in (**a**) but opened both from the top and from the basis; and (**c**) Light capsule created by closing the light-cone both from the top and the basis.

The potential of split-lens structures for the trapping and customized control of particles through photophoretic forces^17^ is highlighted in the following discussion. The first step is the creation of a three-dimensional light structure. To this aim, we analyze a particular example, the use of a light cone for the trapping of particles. As detailed in^29^, a light cone can be generated by using a continuous split-lens configuration (Eq. (1)). The corresponding 3D light distribution is sketched in Fig. 3(a) and experimentally demonstrated in the Results and Discussion Section (see Fig. 4(a)). In particular, by addressing the phase distribution corresponding to a continuous split lens to an SLM (argument in Eq. (1); see the corresponding image pointed by a blue arrow in Fig. 3(a)), a ring of light is created at the focal plane of the lenses (S plane in Fig. 3(a), to a distance *f*_1_ from the SLM plane). Each point of this light ring acts as a new punctual light source, and due to the cylindrical symmetry of the structure, this defines a light cone with its axis parallel to the axial direction. Finally, to a certain propagated plane, interference between light beams coming from the light ring at the S plane occurs. In our particular case, the circular geometry of the light ring leads to a Bessel beam structure to a propagated plane, this beam profile being of interest in different applications^37,38^.

In our case of interest, we focus on the light-cone structure (Fig. 3(a)). We want to note that because the split lens are based on an analytical formulation based on physical parameters of easy interpretation (e.g., Eq. (1)), the geometry of the cone can be easily modified just by tuning these few control parameters, as the focal length of the split lenses or the split-lens sector distances to the center. To better illustrate this fact, we include the inset image in Fig. 3(a). By setting a distance *a* of the split-lens sectors to the center and a focal length *f*_1_, a particular light-cone structure is generated (see inset-image (*i*)). Then, if the distance to the center is reduced to a value *b* < *a*, the length of the light cone is as well linearly reduced with *a* (see inset-image (*ii*)). The spatial position of the cone can also be tuned, for instance, by using a focal length for the split lenses ${f}_{2} > {f}_{1}$ (see inset-image (*iii*)). However, as the cone length is also related with the focal length, the final length is also modified in this third case. In particular, according to geometrical reasons, the length of the generated cone can be written as,

2 $${L}_{cone}=af/{\varphi },$$

where *a* and *f* are the split-lens sectors distance to the center, and their focal length, respectively. The parameter *ϕ* states for the aperture size of the whole lens (i.e., the lens without being split).

Once the control parameters are set at will to design a tailored light cone, the next step is to create a top-opening in the structure, in order to let the microparticles to fall inside the light cone. Methods to generate holes in light bottles were addressed by different authors^28,39^. In our case, this step is conducted as illustrated in Fig. 3(b). To do so, a black triangle is multiplied at the top of the phase distribution to be sent to the SLM (see blue arrow below the SLM in Fig. 3(b)). By doing this, the generated cone has an aperture at the top, from which particles can be introduced into the cavity. Once the particle is captured by the opened cone due to the created photophoretic forces, the light structure must be closed once again to completely trap the particle (see Fig. 3(c)). This is easily conducted just by removing the black triangle from the split lens phase distribution, and thus, recovering the original light-cone structure. Finally, to ensure a stable encapsulation of the particles into the light structure, the left-open side of the cone structure (the open basis at the ring plane; S plane in Fig. 3) must be closed. To that end, a quadratic lens (regular lens) can be multiplexed to the modulator with a focal length *f* < *f*_1_. In such scenario, the regular lens focal spot behaves as a stopper at the cone open basis (i.e., it sets the cap for the optical bottle). At this stage, further manipulation of the spatial position of the particle can be dynamically achieved by modifying the split-lens configuration. For example, the axial position of the particles could also be controlled by tuning the above stated control parameters (see inset image in Fig. 3(a)). Last but not least, if required, the transverse position of the particles could also be controlled by modifying the center coordinate of the split lens structure. We want to note that the above-stated discussion is a proof of concept of the split lens suitability to be applied for particle trapping applications based on photophoretic forces. However, a number of different three-dimensional structures could be generated by using other split-lens configurations, as those leading to optical bottles proposed in^29^.

## Results and Discussion

The capability of trapping and spatially controlling microspheres by using split-lens schemes is experimentally provided in this section. To this aim, customized three-dimensional light structures were created, they being the light-containers where particles are trapped according to photophoretic forces^17^. These light structures were created by addressing split-lens configurations onto a Liquid Crystal on Silicon (LCoS) display. The split-lens concept is reviewed in the Theoretical background Section, so it is taken for granted in this section. The optical arrangement used to experimentally generate the used light-structures is detailed in the Methods Section.

First of all, we demonstrate the capability of creating a three-dimensional light structure and tuning its geometrical properties. This is provided by experimentally generating and manipulating a light-cone structure, being achieved by digitally addressing continuous split lens schemes to the LCoS display (see Theoretical background Section). We chose this particular example (light cone) for the experimental demonstration for the sake of consistency, as such light-structure was used as a proof of concept of the method (for its simplicity and clarity) in the Theoretical background Section (see Fig. 3).

**Figure 5:**
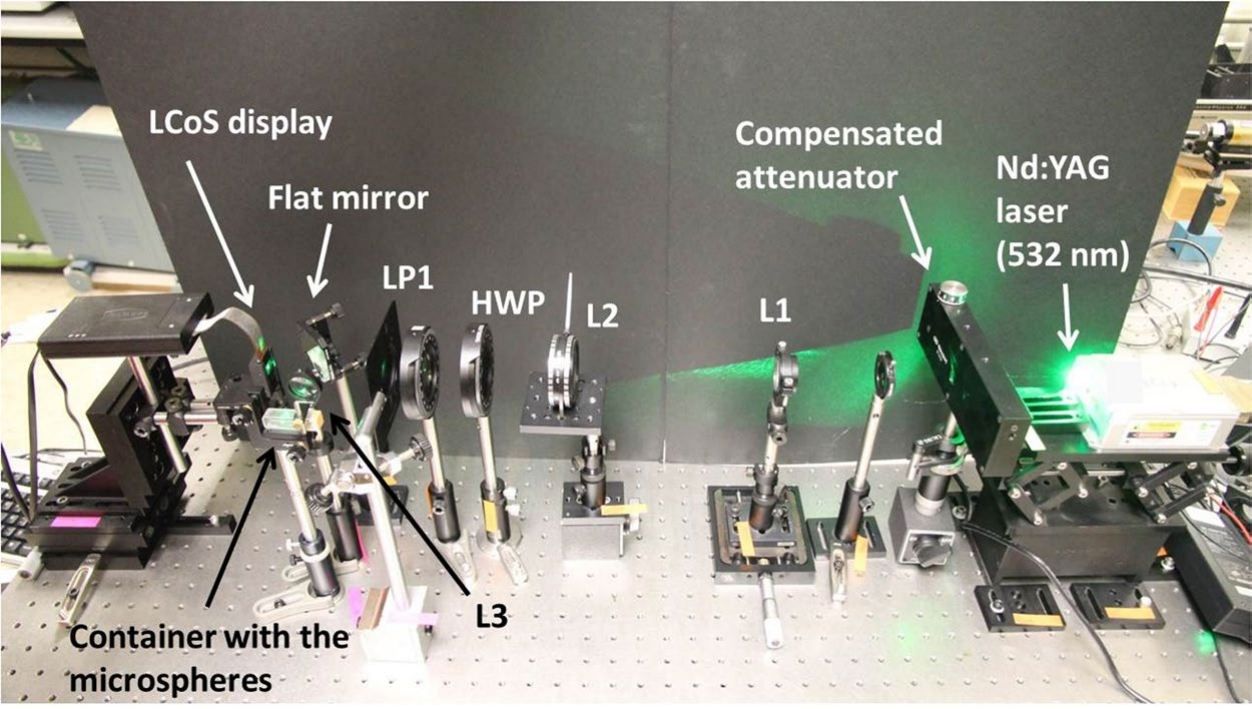
Experimental set-up.

The first experimental example is given in Fig. 4(a), where we show an experimentally generated light cone. The particular control parameters (i.e., parameters set for the continuous split lens) used to generate this structure were: *a* = 1.07 mm, *f* = 370 mm and *ϕ* = 2.07 mm; they referring to the distance between split lens to the coordinate center, the continuous split lens focal length and the lens aperture (a Gaussian beam was illuminating an area of ∼3.3719 mm^2^ at the LCoS screen), respectively. According to Eq. (1) in the Theoretical background Section, by selecting these parameter values, a theoretical light cone with a length of 191 mm is created. However, due to the imaging effect of the convergent lens L3 (at 89 mm from the LCoS display) in the experimental set-up (see Methods Section and Fig. 5), the actual theoretical light cone length is of 5.52 mm. This theoretical result is in agreement with the experimental measures conducted on the generated light cone, where we obtained an experimental length of 6.5 mm, as shown in Fig. 4(a). The experimental length of the cone was obtained by using a CCD camera (7.4 *μm* pixel size) at the reflected arm, mounted in a linear stage able to modify the axial position. Transverse intensity images were then obtained along the light cone axis (i.e., two-dimensional light ring images corresponding to different cone axis positions), with steps of 0.25 mm. From that set of images, the three-dimensional reconstruction of the experimental light structure shown in Fig. 4(a) was obtained by using the ImageJ software. In addition, a visualization of the created 3D structure through the axial direction is also provided in video format (see Supplementary Video 1), where we can see how the light ring created at the focal plane of the split lenses is progressively shrunk as we move forward the cone axis. Note that for the sizes and distances used in our experimental system (CCD pixel size, L3 illuminated area and distance between L3 and imaged cone), the generated cone showed a focal depth of ∼0.5 mm both for the basis and the vertex cone, this leading to an error (∼1 mm) in the determination of the experimental cone length.

To be able to trap particles, we have to be capable of opening the created light-structure from the top, so they can fall in. According to the method detailed in the Theoretical background Section (see Fig. 3), we reconfigured the light cone in Fig. 4(a) by opening it from the top, so particles can be captured into the hollow cone. Resulting experimental opened cone is shown in Fig. 4(b). To create such light-structure, the continuous split lens phase distribution sent to the LCoS display is multiplied by a black-triangle (see inset image in Fig. 3(b)). This black-triangle removes part of the phase pattern, and thus, part of the light-structure is eliminated. When this triangle-function is properly oriented, the above-opening is created. To control the size of the created aperture, we can play with the value set for the angle *α*, being the angle opposite to the black-triangle basis (see inset image in Fig. 3(b)). For the particular case shown in Fig. 4(b), an angle *α* equal to 120 degrees was set. As no other control parameter was modified, the experimental length of the light-cone was not varied. An axial visualization of the experimentally created opened light-cone structure is shown in Supplementary Video 2.

Finally, to capture a particle, once it falls into the cavity of the cone structure, we need to close once again the top-opening aperture. This is easily achieved just by removing the black-triangle function from the phase distribution addressed to the LCoS (see Fig. 3(c)). However, at this point, the light cone basis is still opened, so particles can escape from such aperture. To solve this situation, an extra diffractive element is created, responsible for closing the basis aperture. In particular, a regular diffractive lens with a focal length shorter than that of the continuous lens is simultaneously addressed to the LCoS display (i.e., regular and continuous lenses are multiplexed according to the method given in^40^). Under this scenario, the regular diffractive lens focal spot acts as a light-cork of the light cone, leading to a light capsule (optical bottle). The experimental realization of this closed light structure is shown in Fig. 4(c). Note that light capsule in Fig. 4(c) is both closed from the top and the basis, so particles can be perfectly trapped into the light-structure. Note as well that due to the propagation of the light coming from the current lens focus (light cone cork), a light background is added to the full structure. However, as light coming from the lens focus rapidly diverges, this light background does not affects in terms of particles trapping, as corresponding photophoretic forces are much weaker than forces created by the light cone walls.

Once the generation and control of light structures was provided by analyzing the above-stated light-cone case, we further present the suitability of the method to the trapping of microparticles. What is more, the control of the spatial position of the particles along the axial direction is also provided, so a reconfigurable 3D photophoretic trap is demonstrated. This last feature is granted by modifying the geometrical properties of the light-cone distribution capturing the particles, which is done by dynamically tunning few control parameters of the corresponding split-lens configuration. This stated trapping capability is further provided through different experimental results presented in video-format (Supplementary Movies).

The first experiment performed is shown in Supplementary Video 3. We generated the light cone shown in Fig. 4(a) (with *a* = 1.07 mm, *f* = 370 mm and *ϕ* = 2.07 mm). As above-described, an experimental light cone with a length of *Lexp* = 6.5 mm was created. Then, we placed a transparent container (see Methods Section and Fig. 5) so that the generated light cone fit into the container, in a way that the cone axis and the width of the container are parallel. This transparent box contains carbon microspheres (with sizes in a range of 63–75 *μm*) that are stuck at the container top face due to electrostatic forces. Afterwards, by following the method previously discussed, the light cone is opened from above (see Fig. 4(b)). At this point, by delicately tapping the top of the transparent container, where the particles are stuck, some microsphere particles are unstuck and fall. Therefore, as the cone is opened, some microspheres can be caught by the light structure. Note that the container width is of 10 mm, whereas the created light cone has a length of 6.5 mm, and thus, the light structure occupies a ∼65% of the total length where the stuck particles are spatially distributed. Under this scenario, some of the unstuck particles are lost out of the light structure, but those in the proper spatial region can be trapped in the created opened light cone. This is the case shown in Video 3 (see second 2). However, due to different photophoretic forces at the different cone sides in the axial direction (vertex and basis), the trapped particle moves to the cone open basis side and falls out to the ground. Note that the asymmetric photophoretic forces are related to different light intensities at different cone sides due to the light-cone spatial geometry (there is cylindrical symmetry in a transversal plane but there is not symmetry along the cone axial direction).

A second experiment is provided to show the trapping capability of the system. This is given in Supplementary Video 4. In this case, we conducted the same experiment shown in supplementary Video 3, but now, a regular diffractive lens was addressed to the SLM together with the light-cone structure. To simultaneously address the two phase distributions (diffractive lens and light cone) to the SLM, they were multiplexed into the LCoS display^40^. In particular, the focal length of the regular diffractive-lens (*f*_*lens*_ = 350 mm) was selected to be slightly shorter than the distance between the SLM and the continuous ring plane (i.e., shorter than *f*_*continous*_ = 370 mm). Under such scenario, the focal spot of the regular diffractive lens creates a stopper on the light structure, this meaning that the light cone open side (cone basis) is closed. By using this scheme, the created light capsule prevents particles from escaping through lateral sides. Then, the light cone is opened from above, and a given micro-particle falls into the light-structure. However, as the cone basis is now closed by the diffractive lens effect, the microsphere keeps trapped and stands still at certain axial position (second 6 in the Video 4). To ensure the encapsulation of the microsphere, the light-cone structure is then closed from above, following the method previously detailed (Fig. 3(a–c)). By doing this, we set a light capsule as that in Fig. 4(c) with a particle perfectly trapped inside. In particular, we recorded the particle trapped and kept still over 5 minutes–we removed this 5 minutes-period from the Supplementary Video 4 to prevent it to be excessively long and redundant. Moreover, we also achieved other experiments with trapping-durations larger than 1 hour (until we stopped the experiment). This experimental situation provides the robustness and stability of the created traps.

Last but not least, further control of the micro-particles is also demonstrated in Supplementary Video 4. To a certain moment of the trapping process (after ∼ 5 minutes), we simultaneously modify the focal length of the two multiplexed diffractive lenses (regular lens and continuous split lens) by an amount of 11 mm (i.e., the regular lens focal length increases from *f*_*lens*_ = 350 mm to *f*_*lens*_ = 361 mm and the split lens from *f*_*continous*_ = 370 mm to *f*_*continous*_ = 381 mm). Under these physical conditions (*a* = 1.07 mm, *f*_*continous*_ = 381 mm and *ϕ* = 2.07 mm), the theoretical length of the cone changes from ∼191 mm to ∼196 mm. Again, by taking into account the effect of the imaging lens L3 in the experimental set-up (see Fig. 5), the final calculated cone length is reduced to ∼5.35 mm. This estimation agrees with the experimental measure— ∼5.25 mm (once again, this measure is affected by ∼1 mm error related to the focal depth of the system). What is more, as the split lens focal length *f*_*continous*_ was increased, the spatial position of the closed-light cone is also displaced. This is because the *S* plane in Fig. 3 is shifted and axially drags the full light-capsule structure. In particular, by considering the above-stated control parameters values, the whole cone structure is axially shifted by a positive distance of ∼0.47 mm (∼5% of the full container width). This value was experimentally checked and we obtained an experimental shift of 0.65 ± 0.01 mm. Moreover, as the focal length of the regular lens responsible for closing the cone basis open-side was also corrected, the created trap keeps closed after the spatial displacement. Consequently, the microsphere axial position is modified according to the light cone customized shift. This is shown from second 22 in Video 4, where we observe, as predicted, how the trapped microshpere is displaced to a positive distance. This situation shows the capability of the method to generate photophoretic traps feasible to dynamically modified the 3D position.

Finally, the above-shown experimental results provide the capability of the method to axially control the spatial position of the captured microspheres, but we want to emphasize that the method is valid to arbitrarily modify the spatial position of the micro-particles. For instance, to move particles in the opposite direction to that selected in Video 4, instead of increasing the focal length of the two light structures multiplexed (i.e., the regular and the split lens), as done in Video 4, we only need to reduce them according to distance we want to shift the particle. Moreover, if the transverse position control is of interest as well, our method is also valid for such purpose, just by tunning the central-position of the cone-light structure (i.e., by properly setting the center of the continuous split lens). Therefore, a wide variety of situations can be dynamically achieved by simply tunning the method control parameters.

## Conclusions

We presented an experimental method for the generation of reconfigurable 3D light structures and their use for the precise spatial control of micro-particles through photophoretic force. The generation of the light structures is based on the implementation of split-lens schemes, which allow us to vary some spatial properties of the light-cavities just by modifying some control parameters of simple physical interpretation. In this manuscript, we described the main concepts of the method, detailed the optical scheme used to implement it, and proved the potential of the technique by conducting a series of experiments. In particular, we demonstrated the method capability to create light-cavities, to customize some spatial properties of the created light structures, and to translate their spatial position at will. This capability is experimentally used to capture microspheres, with sizes in a range of 63–75 *μm*, to kept them still in a certain spatial position, and to modify their location in a customized way. The experiments selected to highlight the potential of the method show how the generated structures find application for the implementation of reconfigurable photophoretic traps in three dimensions.

## Methods

In this section we describe the optical set-up used to create the light structures applied for the particles trapping and control we shown in the Results and Discussion Section. The optical set-up is based on the scheme shown in Fig. 3. However, as the system includes a Liquid Crystal on Silicon (LCoS) display, which is a reflective device, the optical scheme in Fig. 3 is adapted to a reflective configuration. The experimental arrangement is shown in Fig. 5. A CW solid-state Laser with a central wavelength of 532 nm was used as a light source. The Laser is a gem 532 Model manufactured by Laser Quantum, with a power ranging from 50 mW to 2 W. After the Laser, we set a Newport 925B Compensated Attenuator, which allow us to control the power of the input beam. This beam illuminates an afocal system, formed by a divergent (L1; *f*_1_ = −100 mm) and a convergent (L2; *f*_2_ = 300 mm) lenses, that expands and collimates the beam setting a beam area of ∼3.3719 mm^2^. Afterwards, we set a half-waveplate (HWP) and a linear polarizer (LP1). By modifying the orientation of the HWP, we control the orientation of the linear polarization exiting from the laser, this allowing us to further regulate the intensity after the LP1 polarizer. The collimated beam illuminates the SLM display. As an SLM, we have used an electrically controlled Parallel Aligned (PA) LCoS display. The PA-LCoS display used is a PLUTO-LCoS display distributed by HOLOEYE. The display is an active matrix reflective device with 1920 × 1080 pixels and 0.72″ diagonal. The pixel pitch is of 8.0 *μ*m and the display has a fill factor of 87%. The PLUTO displays present a reflectivity of 60–75% (according to the model) and diffraction efficiencies of more than 80%. Thus, a total light efficiency of more than 50% per addressable diffractive device is possible. Note that the LP1 polarizer before the SLM is set to 90 degrees of the laboratory vertical, this direction being parallel to the director axis of the LC molecules. This situation ensures a phase-only performance of the LCoS display. At this stage, different phase distributions (Diffractive Optic Elements, DOEs), related to the light spatial structures described in Theoretical background Section, can be addressed to the modulator. The addressed DOEs generate the corresponding digital holograms to a propagated beam. However, as the LCOS display works in reflection, we set an incident angle slightly out of normal-incidence, to ∼2 degrees. Afterwards, a flat mirror is properly placed to steer the reflected beam to a perpendicular optical arm. To a certain distance from the mirror, according to the focal length set for the addressed split-lens based configurations, the desired three-dimensional light structures are generated. At the reflected beam we placed an extra convergent lens (L3; *f*_3_ = 75 mm) that allows us to compact the optical set-up and to image the generated three-dimensional structure to a given axial position with a certain magnification. At that axial position, a transparent container is placed, in a way that the three-dimensional structure fits inside the container. The container used is a UV fused quartz cuvette, with two polished sides, and with dimensions of 45-10-10 mm (distributed by Thorlabs; CV10Q3500). As shown in Fig. 5, the axial direction of the reflected beam (after being reflected on the mirror), is parallel to the width of the container, so the maximum length of the generated light-structures must be smaller than 10 mm.

By using the above-stated optical scheme, the light-cone structures provided in the Results and Discussion Section (see Fig. 4) were generated. Specific details of the created light-structures can be found in this Results Section, which were obtained with the aid of a CCD camera. In particular, the CCD used was a Basler CCD model PIA 1000-60 gm with a resolution of 1000 × 1000 pixels and a pixel size of 7.4 *μm*.

To test the trapping capabilities of the method presented in this work, we used hollow glass microspheres coated with carbon, distributed by Cospheric (HGMS-0.14 63–75 *μm*). They present sizes in a range of 63–75 *μm*, with a density of 0.14 g/*cm*^3^, a typical thermal conductivity of 0.04 W/(m·K), and masses in a range of 18.3–30.9 *ng*. The advantage of using microspheres is the elimination of random body-fixed thermal forces as well as a more accurate evaluation of their mass. The glass microspheres also serve as a model for a transport container, which could be filled with gases, ultrapure materials, or biological substances.

Those microspheres were stitched to the container faces by electrostatic forces. By softly tapping the top of the container, microspheres detached from the top container face, and were trapped into the created light cone. Under the above-described experimental conditions, we successfully trapped microspheres with an input power after the attenuator (see Fig. 5) of approximately 573.26 mW. By taking into account light losses due to reflections on the optical elements and the diffraction on the SLM, a light power of 58.5 mW was measured at the entrance of the transparent container, where the light structure is created. In addition, as shown in the Results and Discussion Section, the trapped microspheres were moved in the axial direction by modifying the *f* parameter of the addressed continuous split lens.

## Electronic supplementary material

Supplementary Video 1

Supplementary Video 2

Supplementary Video 3

Supplementary Video 4

## References

[1] Ashkin A (1997). Optical trapping and manipulation of neutral particles using lasers. Proc. Nat. Acad. Sci..

[2] Mitri F (2017). Self-bending scalar and vector bottle sheets. JOSA A.

[3] Gong, Z., Pan, Y.-L., Videen, G. & Wang, C. Optical trapping and manipulation of single particles in air: Principles, technical details, and applications. *J. Quant. Spectrosc. Radiat. Transf*. (2018).

[4] Grier DG (2003). A revolution in optical manipulation. Nat..

[5] Mitri F (2015). Acoustical pulling force of a limited-diffracting annular beam centered on a sphere. IEEE Trans. Ultrason. Ferroelectr. Freq. Control..

[6] Mitri F (2017). Negative optical radiation force and spin torques on subwavelength prolate and oblate spheroids in fractional bessel-gauss pincers light-sheets. J. Opt. Soc. Am. A.

[7] Maunz P (2004). Cavity cooling of a single atom. Nat..

[8] Barry JF, McCarron DJ, Norrgard EB, Steinecker MH, DeMille D (2014). Magneto-optical trapping of a diatomic molecule. Nat..

[9] Chan J (2011). Laser cooling of a nanomechanical oscillator into its quantum ground state. Nat..

[10] Asenbaum P, Kuhn S, Nimmrichter S, Sezer U, Arndt M (2013). Cavity cooling of free silicon nanoparticles in high vacuum. Nat. Commun..

[11] Juan ML (2017). Cooperatively enhanced dipole forces from artificial atoms in trapped nanodiamonds. Nat. Phys..

[12] Ashkin A, Dziedzic JM, Yamane T (1987). Optical trapping and manipulation of single cells using infrared laser beams. Nat..

[13] Čižmár T, Mazilu M, Dholakia K (2010). *In situ* wavefront correction and its application to micromanipulation. Nat. Photon..

[14] McGloin D, Spalding G, Melville H, Sibbett W, Dholakia K (2003). Applications of spatial light modulators in atom optics. Opt. Express.

[15] Woerdemann M, Alpmann C, Esseling M, Denz C (2013). Advanced optical trapping by complex beam shaping. Laser & Photonics Rev..

[16] Shvedov VG (2010). Selective trapping of multiple particles by volume speckle field. Opt. Express.

[17] Jovanovic O (2009). Photophoresis: Light induced motion of particles suspended in gas. J. Quant. Spectrosc. Radiat. Transf..

[18] Esseling M, Rose P, Alpmann C, Denz C (2012). Photophoretic trampoline-interaction of single airborne absorbing droplets with light. Appl. Phys. Lett..

[19] Eckerskorn N (2015). Optically induced forces imposed in an optical funnel on a stream of particles in air or vacuum. Phys. Rev. Appl..

[20] Arlt J, Padgett MJ (2000). Generation of a beam with a dark focus surrounded by regions of higher intensity: the optical bottle beam. Opt. Lett..

[21] Desyatnikov AS, Shvedov VG, Rode AV, Krolikowski W, Kivshar YS (2009). Photophoretic manipulation of absorbing aerosol particles with vortex beams: theory versus experiment. Opt. Express.

[22] Shvedov VG (2010). Giant optical manipulation. Phys. Rev. Lett..

[23] Shvedov VG, Hnatovsky C, Rode AV, Krolikowski W (2011). Robust trapping and manipulation of airborne particles with a bottle beam. Opt. Express.

[24] Shvedov V, Davoyan AR, Hnatovsky C, Engheta N, Krolikowski W (2014). A long-range polarization-controlled optical tractor beam. Nat. Photon..

[25] Kovalev AA, Kotlyar VV, Porflrev AP (2016). Optical trapping and moving of microparticles by using asymmetrical laguerre-gaussian beams. Opt. Lett..

[26] Shvedov VG, Hnatovsky C, Eckerskorn N, Rode AV, Krolikowski W (2012). Polarization-sensitive photophoresis. Appl. Phys. Lett..

[27] Smalley DE (2018). A photophoretic-trap volumetric display. Nat..

[28] Turpin A (2013). Optical vault: A reconfigurable bottle beam based on conical refraction of light. Opt. Express.

[29] Lizana A (2016). Shaping light with split lens configurations. J. Opt..

[30] Billet, F. Sur un principe d’optique géométrique et sur son application á plusieurs questions et á divers appareils. *mémoires de l’Académie de Dijon* 1–36 (1854).

[31] Born, M. & Wolf, E. *Principles of Optics: electromagnetic theory of propagation, interferences and diffraction of light* (Pergamon Press, 1984).

[32] Cofré A (2017). Dual polarization split lenses. Opt. Express.

[33] Cofré A (2017). Quantitative performance of a polarization diffraction grating polarimeter encoded onto two liquid-crystal-on-silicon displays. Opt. Laser Technol..

[34] Cheng C, Chern J (2010). Symmetry property of a generalized billet’s n-split lens. Opt. Commun..

[35] Cheng C, Chern J (2010). Quasi bessel beam by billet’s n-split lens. Opt. Commun..

[36] Zhang Z, Z. Y., Chu D (2014). Fundamentals of phase-only liquid crystal on silicon (lcos) devices. Light. Sci. Appl..

[37] Planchon A (2011). Rapid three-dimensional isotropic imaging of living cells using bessel beam plane illumination. Nat. Methods.

[38] Chu X (2015). Generating a bessel-gaussian beam for the application in optical engineering. Sci. Rep..

[39] Li X (2018). Controllable mode transformation in perfect optical vortices. Opt. Express.

[40] Campos J (2006). Multiplexed lenses written onto a liquid crystal display to increase depth of focus. Proc.SPIE.

